# Supplementary material for: Using machine learning to predict protein–protein interactions between a zombie ant fungus and its carpenter ant host
Source: Sci Rep. 2023 Aug 24;13:13821. doi: 10.1038/s41598-023-40764-8 (PMC10449854; doi:10.1038/s41598-023-40764-8)
Supplement: Supplementary file 1 — Supplementary Legends. [file 41598_2023_40764_MOESM1_ESM.docx]

**Supplementary Information**

**Discussion S1:** A Word document briefly describing preliminary analysis of transmembrane fungal proteins.

**Figure S1:** Filtering aspecific PPIs and host proteins shared between *Ophiocordyceps* and alternative fungi.

**Figure S2:** D-SCRIPT edge values for tested protein pairings between extracellular fungal proteins and the ant proteome.

**Table S1:** Aspecific fungi and *Ophiocordyceps* PPI overview.

**File S1:** *Ophiocordyceps*-*Camponotus* PPI protein and enrichment data in a single Excel file with multiple tabs.

**File S2:** Aspecific fungi PPI data in a single Excel file with multiple tabs.
